# Prediction of new vertebral compression fracture within 3 years after percutaneous vertebroplasty for osteoporotic vertebral compression fracture: Establishment and validation of a nomogram prediction model

**DOI:** 10.1371/journal.pone.0303385

**Published:** 2024-05-21

**Authors:** Mingxi Nie, Zefu Chen, Liang Shi, HongXia Cao, Lei Xu

**Affiliations:** 1 Department of Emergency, Xiangyang No. 1 People’s Hospital, Hubei University of Medicine, Shiyan City, Hubei Province, China; 2 Department of Orthopedics, Xiangyang No. 1 People’s Hospital, Hubei University of Medicine, Shiyan City, Hubei Province, China; 3 Department of Rehabilitation Medicine, Xiangyang No. 1 People’s Hospital, Hubei University of Medicine, Shiyan City, Hubei Province, China; Duke University Medical Center: Duke University Hospital, UNITED STATES

## Abstract

New vertebral compression fractures (NVCF) are common in patients with osteoporotic vertebral compression fractures (OVCF) who have undergone percutaneous vertebroplasty (PVP). We sought to develop a nomogram prediction model for better identification and prevention of NVCF within 3 years after PVP in patients with OVCF. The demographic, clinical, and imaging data of patients who underwent PVP for OVCF between January 2010 and December 2019 were reviewed. Multivariate logistic regression analysis was used to screen for risk factors for NVCF within 3 years after PVP. A nomogram prediction model was then developed and validated to visually predict NVCF. The samples in the model were randomly divided into training and validation sets at a ratio of 7:3. Twenty-seven percent of patients experienced NVCF in other segments within 3 years after PVP. Older age, lower bone mineral density (BMD), smoking, lack of anti-osteoporosis therapy, and postoperative trauma were risk factors for NVCF. The area under the receiver operating characteristic curve suggested good discrimination of this model: training set (0.781, 95% confidence interval: 0.731–0.831) and validation set (0.786, 95% confidence interval: 0.708–0.863). The calibration curve suggested good prediction accuracy between the actual and predicted probabilities in the training and validation sets. The DCA results suggested that, when the probability thresholds were 0.0452–08394 and 0.0336–0.7262 in the training and validation set, respectively, patients can benefit from using this model to predict NVCF within 3 years after PVP. In conclusion, this nomogram prediction model that included five risk factors (older age, lower BMD, smoking, postoperative minor trauma, and lack of anti-osteoporosis treatment can effectively predict NVCF within 3 years after PVP. Postoperative smoking cessation, standard anti-osteoporosis treatment, and reduction in incidental minor trauma are necessary and effective means of reducing the incidence of NVCF.

## Introduction

With the inevitable increase in the aging global population, osteoporosis is becoming more common [1,2]. In the USA, an investigation of 1948 men and 1639 women found that 16% of men and 29.9% of women aged > 50 years had osteoporosis [3]. In China, a multicenter study involving 7042 subjects from 10 centers on the mainland found a prevalence of 10.4% and 31.3% in men and women aged > 50 years, respectively [4]. Osteoporotic vertebral compression fracture (OVCF) is common in the elderly [5–7]. Kwok et al. found an OVCF prevalence of 5.0% and 12.1% in men and women aged > 65 years, respectively. In addition, the prevalence rate increased from 2.9% in men aged 65–69 years to 11.4% in those aged > 80 years, and from 6.1% to 22.6% in women in the same age groups [8]. A recent systematic review and meta-analysis by Fan et al. found that the prevalence of osteoporosis in spinal surgery patients older than 50 years was 34.2% (2958 individuals). Among them, in the age groups of 50–59, 50–69, and 70–79, the prevalence of osteoporosis for females were 27.8%, 60.4%, and 75.4%, respectively, and for males were 18.9%, 17.4%, and 26.1% [9]. In addition, a recent nationwide cohort study of 291,203 cases from South Korea found that the mortality rate of patients with OVCF was 1.22 times that of patients without OVCF after controlling for differences in sex and age [10].

Generally, OVCF can be treated conservatively or with minimally invasive surgery, such as percutaneous vertebroplasty (PVP) [11–13]. PVP is widely used because of patients’ faster pain recovery and better immediate- and short-term prognoses compared with conservative treatment [11,12]. However, the OVCF treatment is not a once-off approach. According to previous reports, the probability of new vertebral fractures or original vertebral refractures after OVCF ranges from 9.3% to 38.4% [14–24]. Previous studies have reported risk factors for new vertebral compression fractures (NVCF) after OVCF, including higher body mass index (BMI), higher initial symptomatic fractures, lower bone mineral density (BMD), lower computer tomography (CT) Hounsfield unit (HU) value, older age, female sex, diabetes, and intravertebral cleft [14–24]. Although many risk factors have been identified, they cannot be visualized to obtain a relatively accurate probability of new fractures based on patient characteristics. Recently, some studies have developed clinical predictive models to predict NVCF after PVP or percutaneous kyphoplasty in patients with OVCF [25–28]. However, in their study, the follow-up time of patients was short (within 2 years after PVP) [25] or did not specify the follow-up time [26–28], which may not be enough to predict the longer-term outcome of OVCF patients or did not provide the NVCF rate of patients in a certain duration. Furthermore, in addition to the clinical and radiological factors commonly mentioned in previous studies, some important new clinical or radiological parameters, such as postoperative smoking, additional postoperative minor trauma, lack of postoperative anti-osteoporosis treatment, and Kümmell disease of the primary fractured vertebra, were also considered in this study. Additionally, based on potential risk factors, we developed a nomogram model to visually predict NVCF 3 years after PVP in patients with OVCF.

## Material and methods

### Participants

This study reviewed prospectively collected data including the demographic, clinical, and imaging data of patients undergoing PVP surgery for OVCF at one spine center from January 1, 2010 to December 31, 2019. This study has been reviewed and approved by the Ethics Committee of Xiangyang No.1 People’s Hospital, Hubei University of Medicine. Due to the retrospective nature of this study, all patients were exempted from signing informed consent forms. The data were accessed for research purposes between January 2022 and December 2022. The authors had access to information that could identify individual participants during or after data collection. The patients were divided into two groups: the NVCF group and the group without NVCF within 3 years after PVP.

Inclusion criteria: 1). Patients aged ≥50 years. 2). Patients undergoing PVP for OVCF. 2). Patients with complete data. 3). Patients with or without NVCF beyond the level of the primary fractured vertebra 3 years after PVP. 4). Patients with follow-up time of 3 years or more.

Exclusion criteria: 1). Patients with OVCF treated conservatively or with other internal fixation procedures. 2). Patients with pathological fractures, such as those due to spinal infections or spinal tumors. 3). Non-OVCF patients included those with spinal fractures caused by high fall injuries or other high violence injuries. 4). Patients were excluded if they had a second fracture of primary fractured vertebra.

### Demographic data

Demographic data were collected and tallied by an independent follow-up person who was blinded to the patient grouping for this study. Demographics included sex; age; diagnosis; BMI; BMD; history of diabetes mellitus, smoking, drinking, postoperative minor trauma, and anti-osteoporotic measures. The patient’s information such as sex, age, diagnosis, BMI, BMD, and history of diabetes mellitus comes from the hospital’s medical record system. The BMD value was obtained from the results of the lumbar spine examination using dual-energy X-ray absorptiometry. The smoking and drinking history of the patient was known by the follow-up staff through questioning the patient. Smoking was defined as a history of smoking after the PVP. Drinking history was defined as a history of drinking after PVP. The average consumption of at least one pack of cigarettes (20 cigarettes) per week was considered to be a history of smoking, and the average consumption of at least one or more drinks per week was considered to be a history of drinking. A history of postoperative minor trauma was defined as a minor traumatic event after PVP, such as accidental falling, slipping, or sudden weight bearing. Anti-osteoporosis protocols were formulated by orthopedic surgeons and endocrinologists. A lack of anti-osteoporotic measures was defined as the failure to receive anti-osteoporotic treatment according to medical advice.

### Radiological data

Radiological data included the location of the primary fractured vertebra/e, number of segments of the primary fractured vertebrae, and the presence or absence of Kümmell disease in the primary fractured vertebra/e (intravertebral left). A one-segment fracture was defined as a single primary fractured vertebra, while multi-segment fractures were defined as primary fractured vertebrae exceeding one segment. Additionally, the patient is classified as thoracic OVCF (T1-T12), lumbar OVCF (L1-L5), and thoracic combined with lumbar OVCF according to the location of the patient’s initial fracture.

### Grouping

The patients were divided into an NVCF and a non-NVCF group based on whether they had experienced NVCF within 3 years after PVP. Patients with NVCF had symptoms such as back and leg pain, as well as corresponding imaging features on spinal X radiography, CT, and magnetic resonance imaging (MRI). Only patients with symptoms associated with NVCF and confirmed by imaging, especially fresh OVCF confirmed by MRI, were included in the NVCF group, otherwise they were included in the non-NVCF group. Patients who experienced a second fracture in the primary fractured segment, but not in the new vertebral segment, were excluded from the study.

### Statistical method

The student’s t-test was used to compare the age, BMI, and BMD of the two groups, and the chi-square or Fisher’s exact test was used to compare sex; history of diabetes, smoking, alcohol intake, and postoperative minor trauma; multisegment fractures; Kümmell disease; and lack of anti-osteoporosis measures between the two groups. Then, parameters with significant differences between the two groups were included in the univariate and multivariate logistic regression analyses. Finally, a nomogram prediction model was developed using the risk factors screened by multivariate logistic regression analysis to predict NVCF within 3 years after PVP for OVCF. The samples in this model were randomly divided into a training and a validation set at a ratio of 7:3. The Receiver operating characteristic (ROC) curve were adopted to evaluate the discrimination of this model. The calibration curve were adopted to evaluate the calibration of this model. The decision curve analysis (DCA) were adopted to evaluate the clinical value of the model. SPSS.25 software (IBM Corp., Armonk, NY, USA) was used for statistical analysis. The R software (version 4.2.1) was used to build and validate the nomogram prediction model.

### Ethics

This study was reviewed and approved by the Ethics Committee of Xiangyang No.1 People’s Hospital, Hubei University of Medicine. All patients waived the need to sign the informed consent form, and the data were analyzed anonymously.

## Results

**Fig 1:**
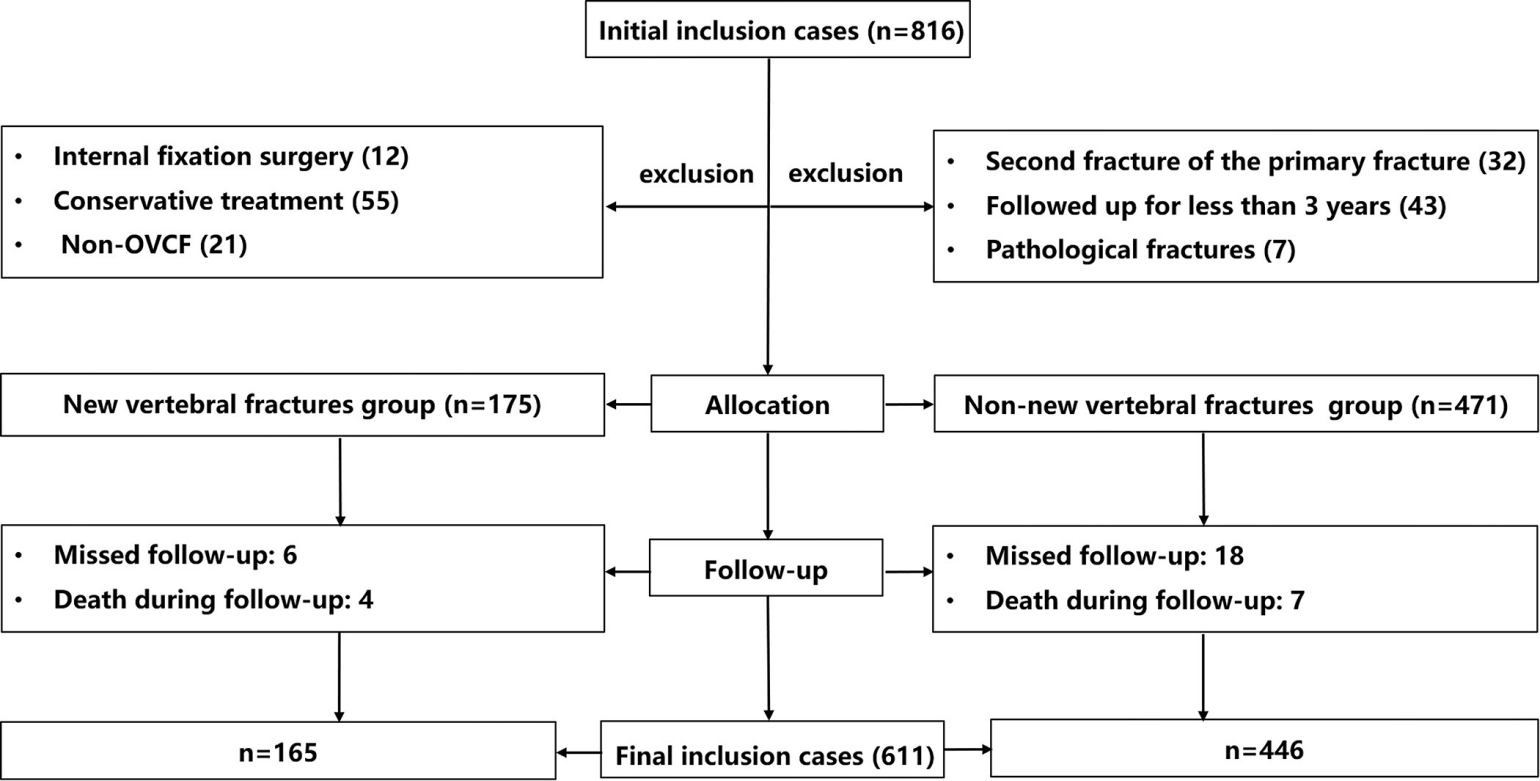
The inclusion and exclusion procedures of this study.

The criteria for inclusion and exclusion are illustrated in Fig 1. The study initially included 816 patients. After excluding 12 internal fixation surgeries, 55 cases with conservative treatments 21 cases of non-OVCF, 32 cases of refracture of the primary fractured vertebra, 43 cases with follow-up of < 3 years, and seven cases of pathological fractures (five cases of spinal infection and two of spinal tumors), we included 175 cases in the NVCF group and 471 cases in the non-NVCF group. During the follow-up period, 24 patients with missed follow-ups and 11 patients who died were excluded. Ultimately, 611 patients (229 males and 382 females) were included in this study. Among them, 165 patients experienced NVCFs within 3 years after PVP, whereas 446 patients did not. The demographic, clinical, and imaging results of the two groups are shown in Table 1. There were no significant differences in sex, BMI, proportion of diabetics, sites of VOCF or those with a history of alcohol intake between the two groups. However, the mean age and BMD were significantly higher in the NVCF than in the non-NVCF group. In addition, the proportions of patients who had higher age, smoked, had Kümmell disease, postoperative minor trauma, and lack of anti-osteoporosis treatment were significantly higher in the NVCF than in the non-NCVF group.

**Table 1:** Patients’ clinical parameters of the two groups.

Subgroup	Non-NVCF group (446)	NVCF group (N = 165)	P value
Gender			0.362
Man	172(38.6%)	57(34.5%)	
Woman	274(61.4%)	108(65.5%)	
Age (year)	68.1±6.5	72.8±7.3	<0.001
BMD (T value)	-3.3±0.6	-3.6±0.7	<0.001
BMI (kg/m2)	23.7±2.7	23.4±2.8	0.318
Sites of OVCF			0.454
Thoracic OVCF	177(39.7%)	61(37.0%)	
Lumbar OVCF	254(57.0%)	95(57.6%)	
Thoracic combined with lumbar OVCF	15(3.4%)	9(5.5%)	
Smoking	63(14.1%)	40(24.2%)	0.003
Drinking	62(13.9%)	30(18.2%)	0.496
Diabetes	52(11.7%)	26(15.8%)	0.178
Kümmell disease	69(15.5%)	43(26.1%)	0.003
Postoperative minor trauma	48(10.8%)	41(24.8%)	<0.001
Multisegment fractures	55(12.3%)	36(21.8%)	0.003
Lack of anti-osteoporosis	87(19.5%)	58(35.2%)	<0.001

NVCF: new vertebral compression fracture, BMD: bone mineral density, BMI: body mass index.

The univariate and multivariate logistic regression analyses of new fractures are shown in Table 2. Univariate logistic regression analysis revealed that older age, higher BMD and higher proportions of smoking, Kümmell disease, postoperative minor trauma, multisegment fractures, and lack of anti-osteoporosis treatment were associated with NVCF. However, multivariate logistic regression analysis suggested that older age (p<0.001, OR = 1.084, 95%CI: 1.052–1.117), BMD (p = 0.016, OR = 0.661, 95%CI: 0.472–0.925), smoking (p = 0.024, OR = 1.761, 95%CI: 1.078–2.878), postoperative minor trauma (p = 0.001, OR = 2.281, 95%CI: 1.378–3.781), and lack of anti-osteoporosis treatment (p = 0.001, OR = 2.214, 95%CI: 1.389–3.303) were risk factors for NVCF after PVP in patients with OVCF. This indicates that after excluding the interfering factors (Kümmell disease, multisegment fractures), the probability of developing NVCF increases by approximately 8.4% for every 1 year of age increase in patients. The probability of NVCF increases by approximately 33.9% for every 1 unit decrease in BMD. In addition, the probability of developing NVCF in patients who smoke after PVP surgery is approximately 1.761 times higher than in non-smoking patients. The probability of NVCF occurring in patients with postoperative minor traumas is approximately 2.281 times higher than that of patients without postoperative minor traumas. The probability of developing NVCF in patients without postoperative anti-osteoporosis treatment is approximately 2.214 times higher than that in patients with postoperative anti-osteoporosis treatment.

**Table 2:** Univariate and multivariate logistic regression analysis of NVCF.

Variables	Univariate logistic regression analysis			Multivariate logistic regression analysis		
	P value	OR	95% CI	P	OR	95%CI
Age	<0.001	1.098	1.070–1.127	<0.001	1.084	1.052–1.117
BMD	<0.001	0.445	0.332–0.598	0.016	0.661	0.472–0.925
Smoking	0.003	1.945	1.247–3.035	0.024	1.761	1.078–2.878
Kümmell disease	0.003	1.926	1.250–2.967	0.805	0.936	0.554–1.582
Postoperative minor trauma	<0.001	2.742	1.726–4.356	0.001	2.281	1.378–3.781
Multisegment fractures	0.004	1.984	1.248–3.158	0.096	1.566	0.923–2.658
Lack of anti-osteoporosis	<0.001	2.237	1.505–3.324	0.001	2.214	1.389–3.303

NVCF: new vertebral compression fracture, BMD: bone mineral density.

**Fig 2:**
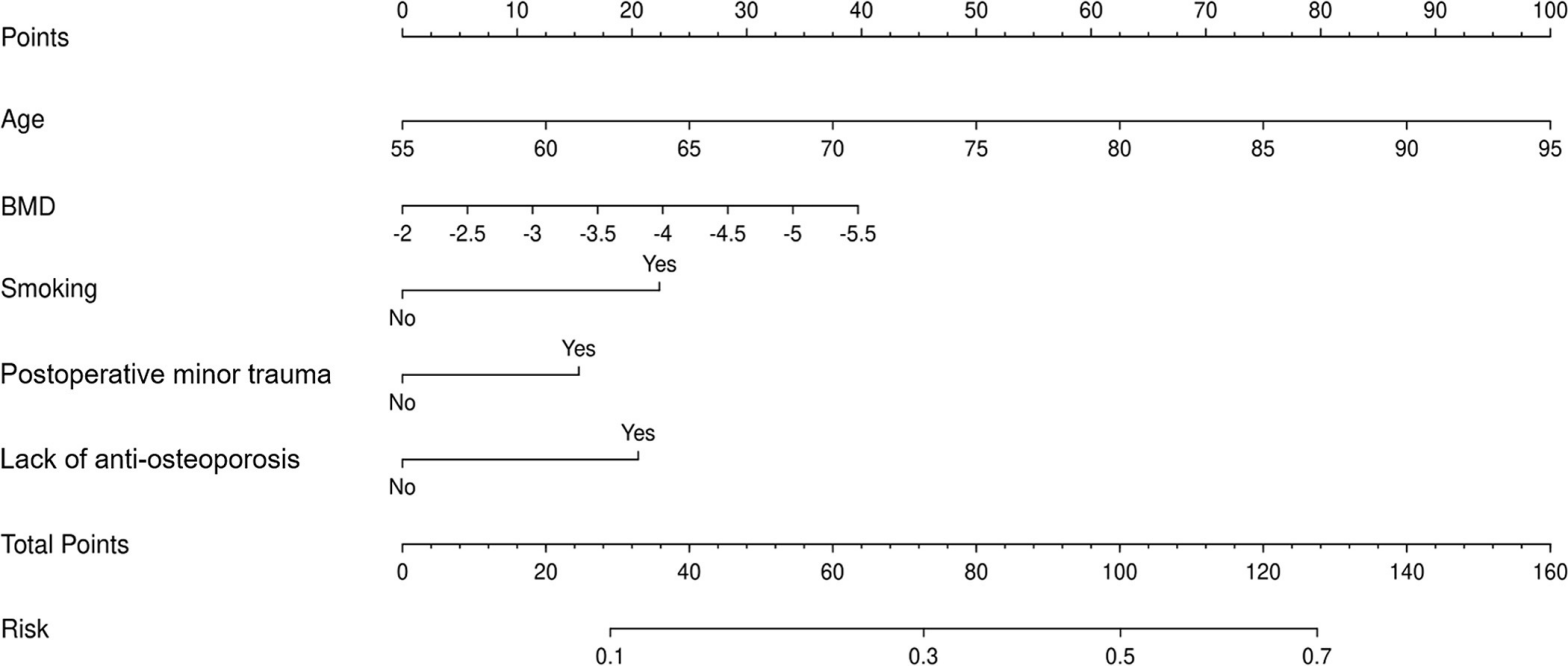
The nomogram model to predict recurrent vertebral fractures within 3 years after PVP.

**Fig 3:**
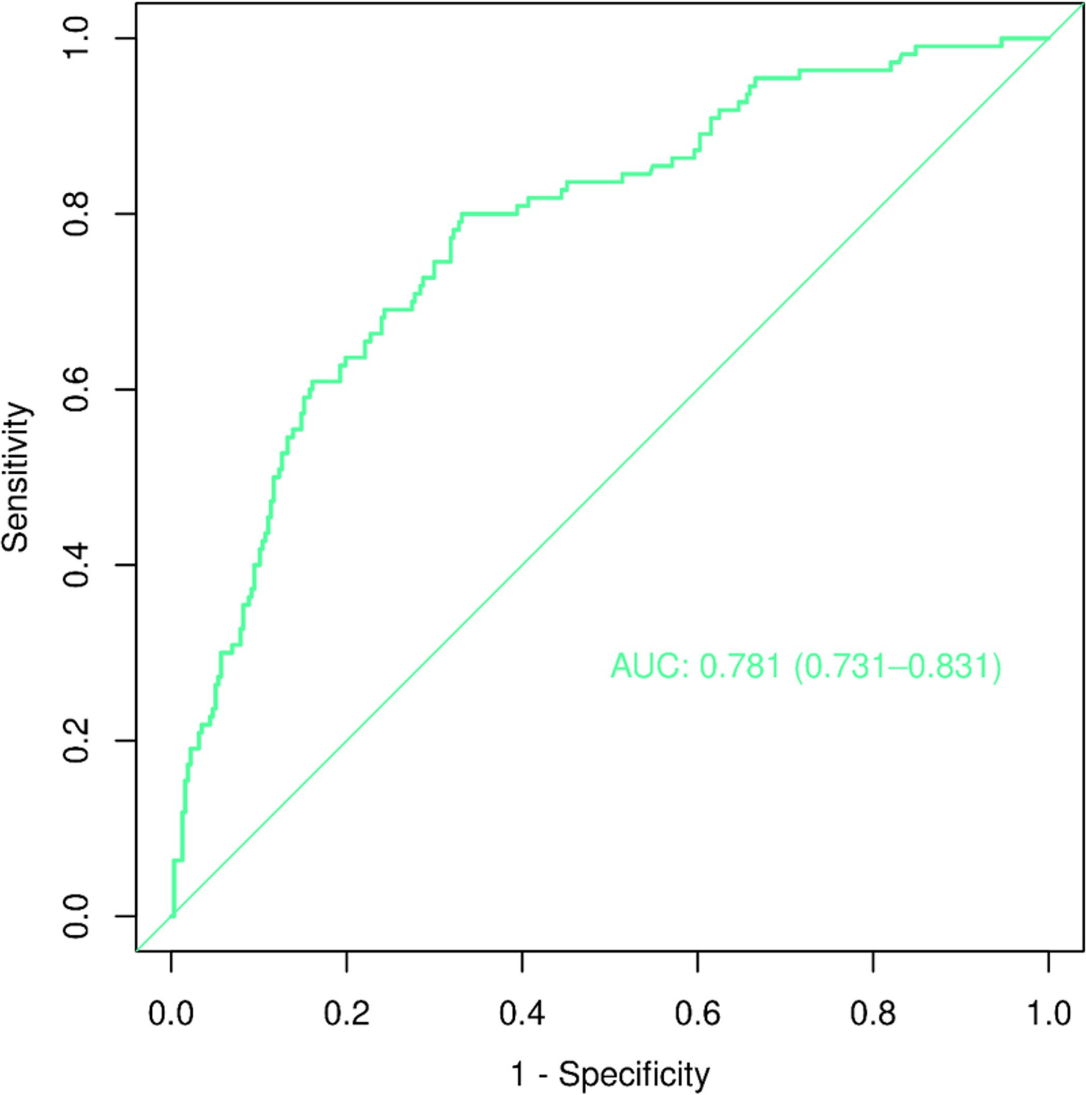
The area under the curve of the receiver operating characteristic curve of the training set (Fig 3) and validation set (Fig 4). The calibration curve of the training set (Fig 5) and validation set (Fig 6), respectively. The decision curve analysis of the training set (Fig 7) and validation set (Fig 8).

**Fig 4:**
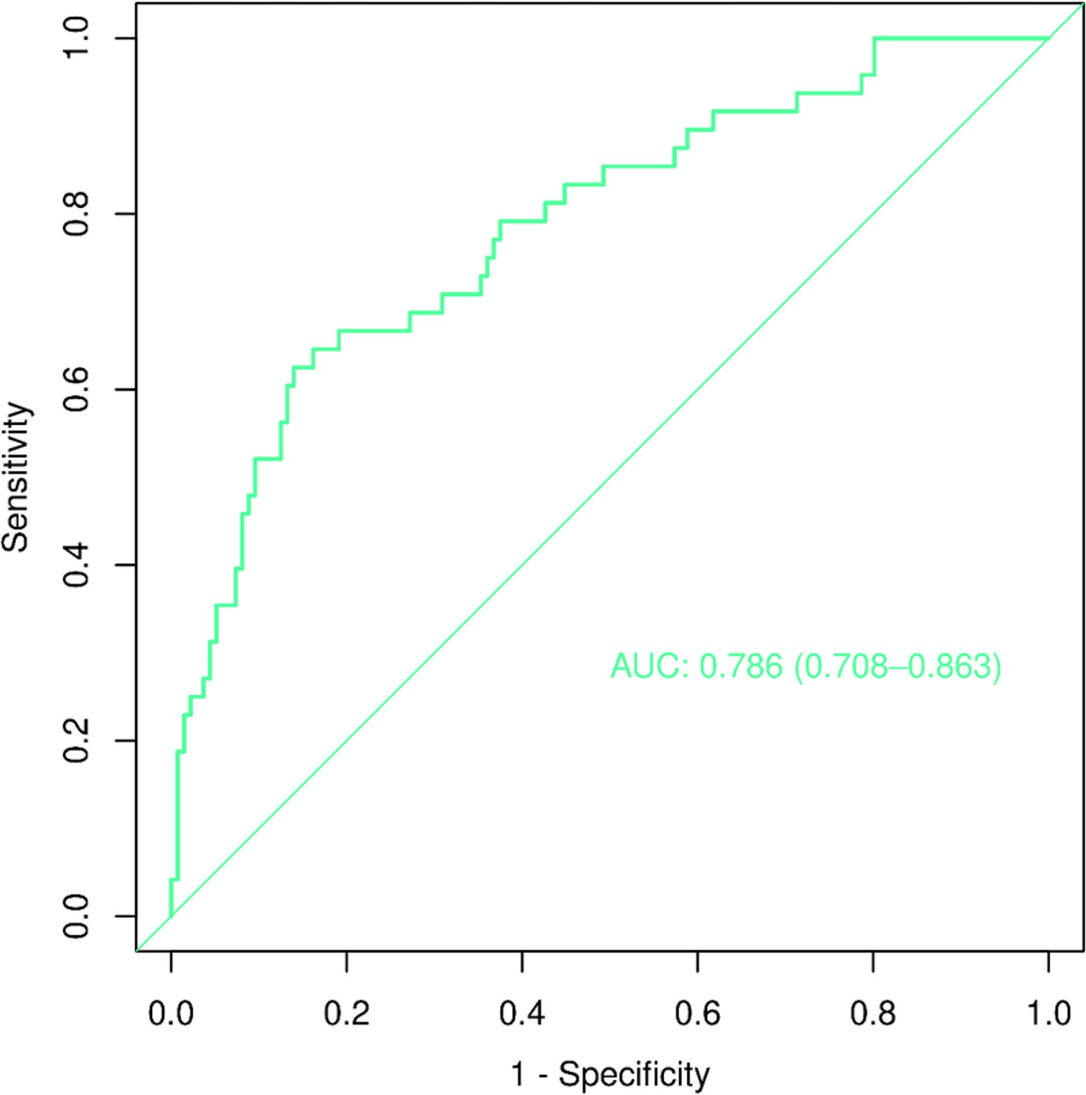


**Fig 5:**
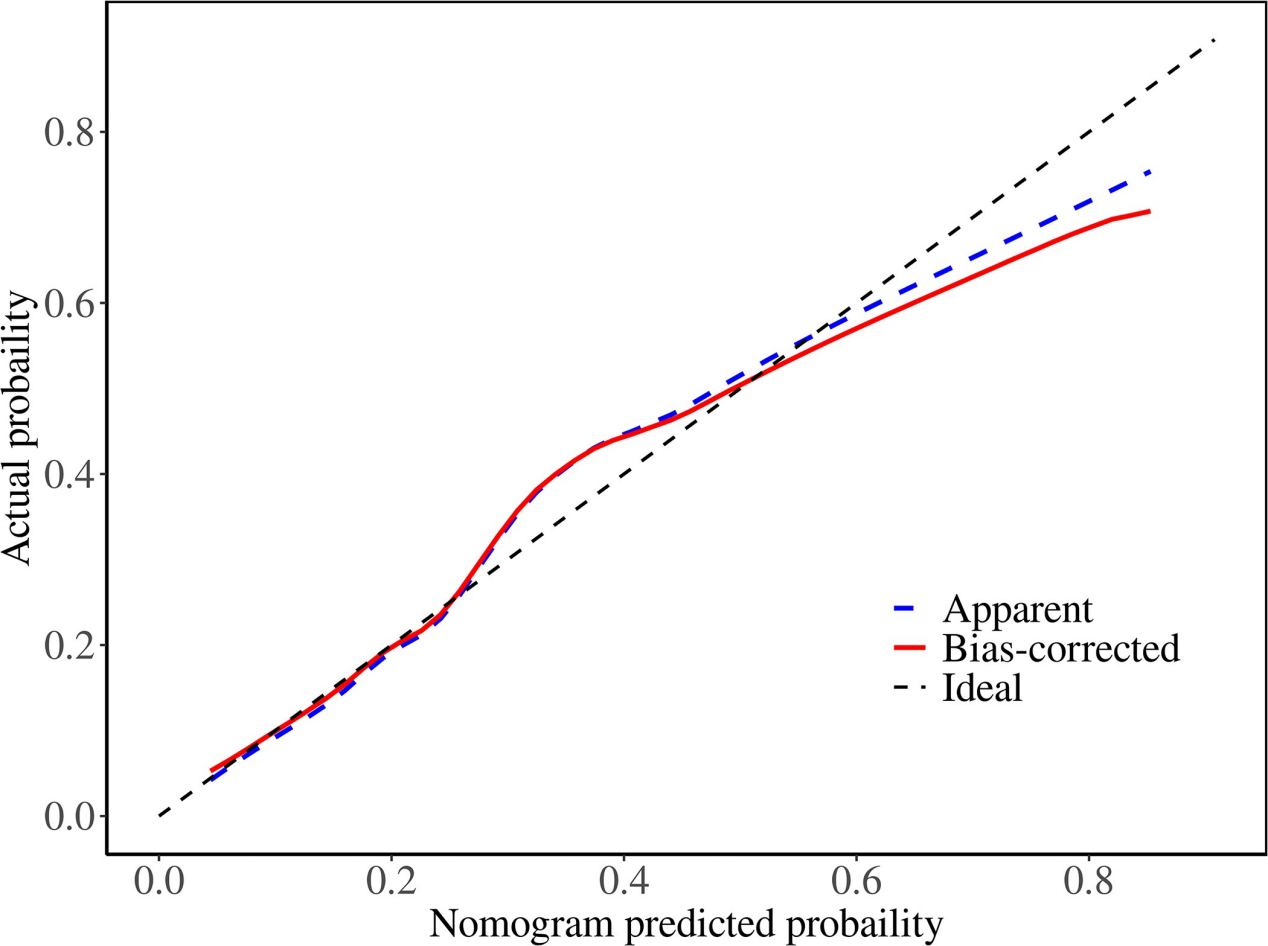


**Fig 6:**
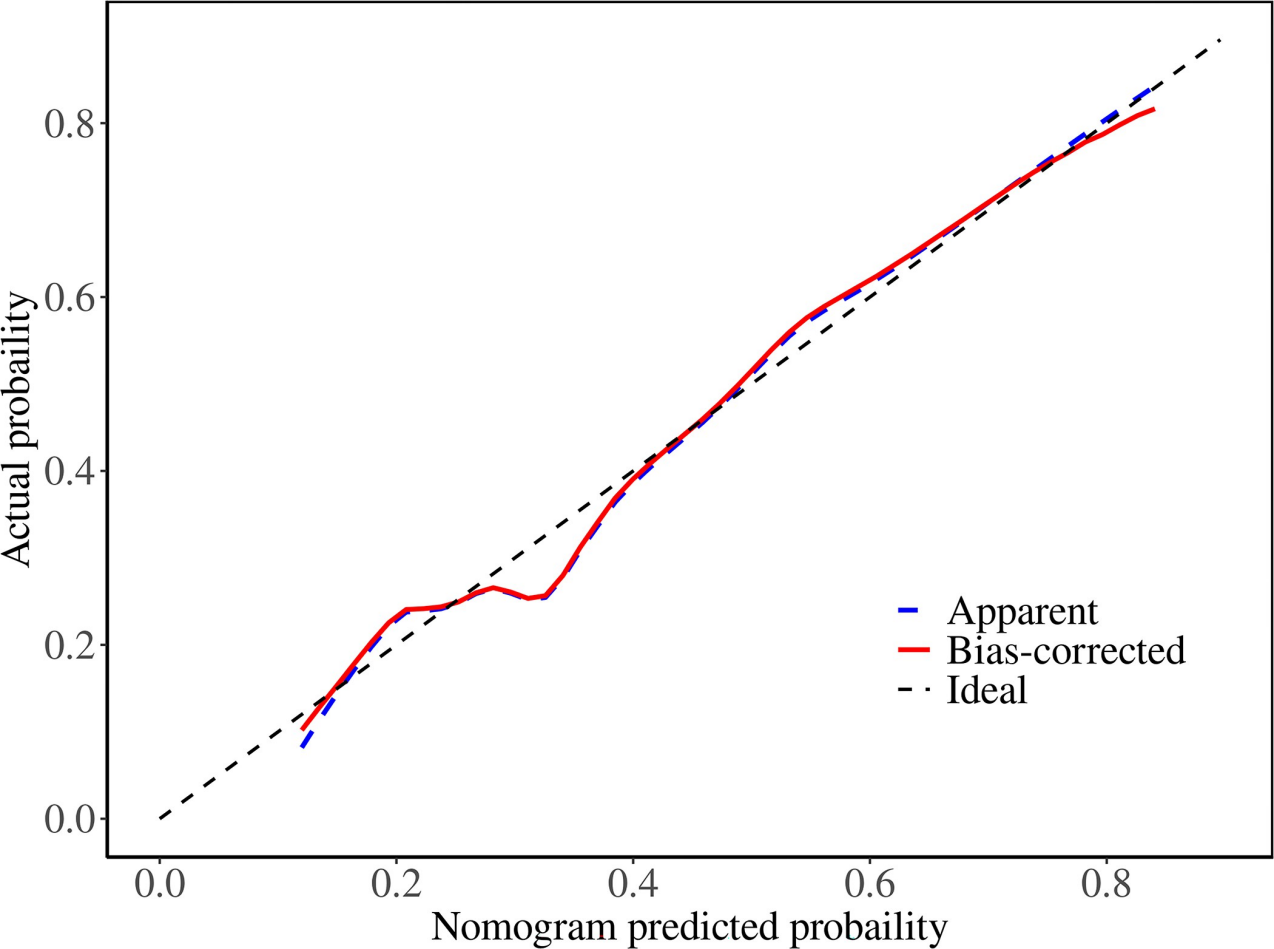


**Fig 7:**
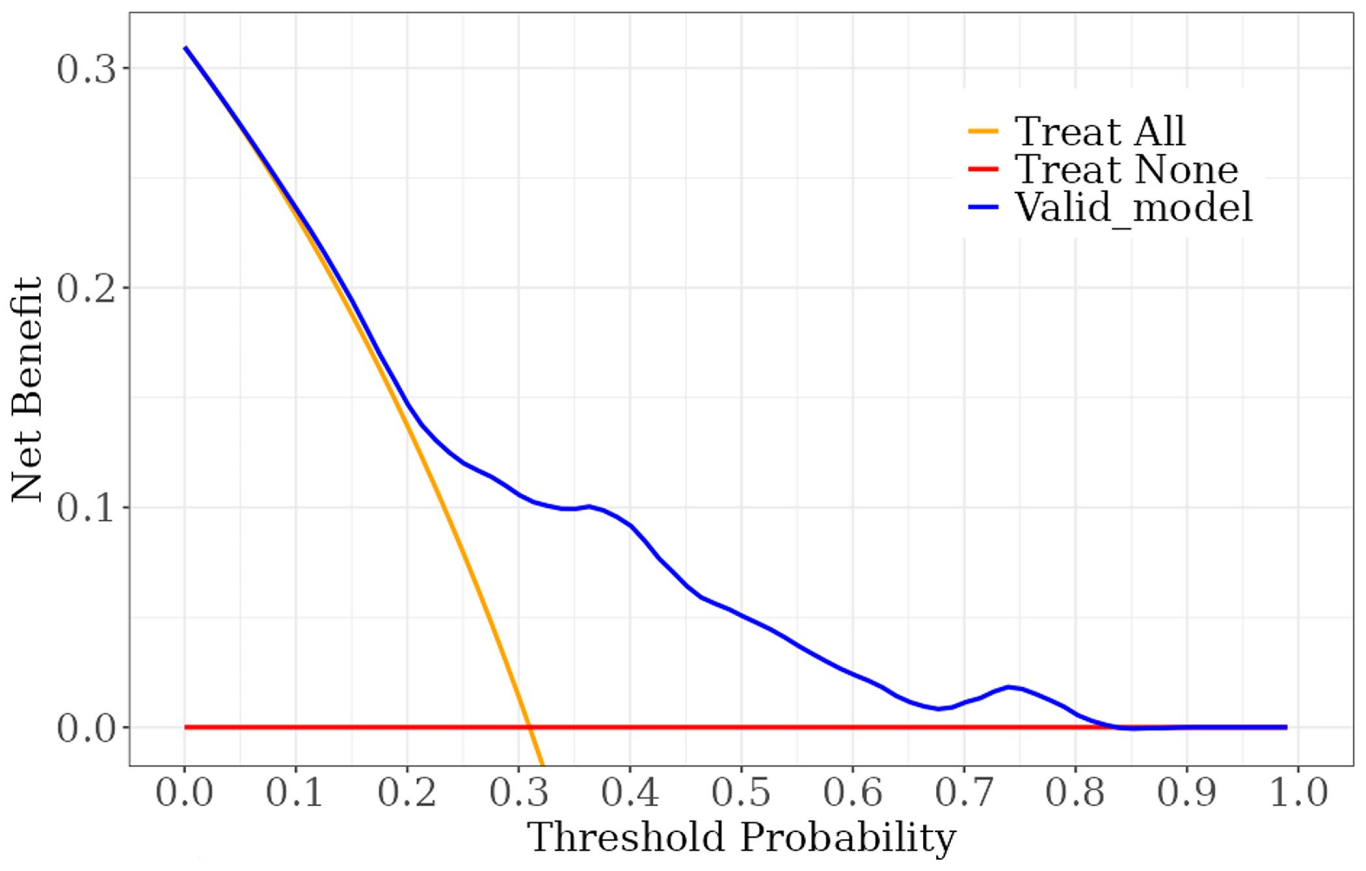


**Fig 8:**
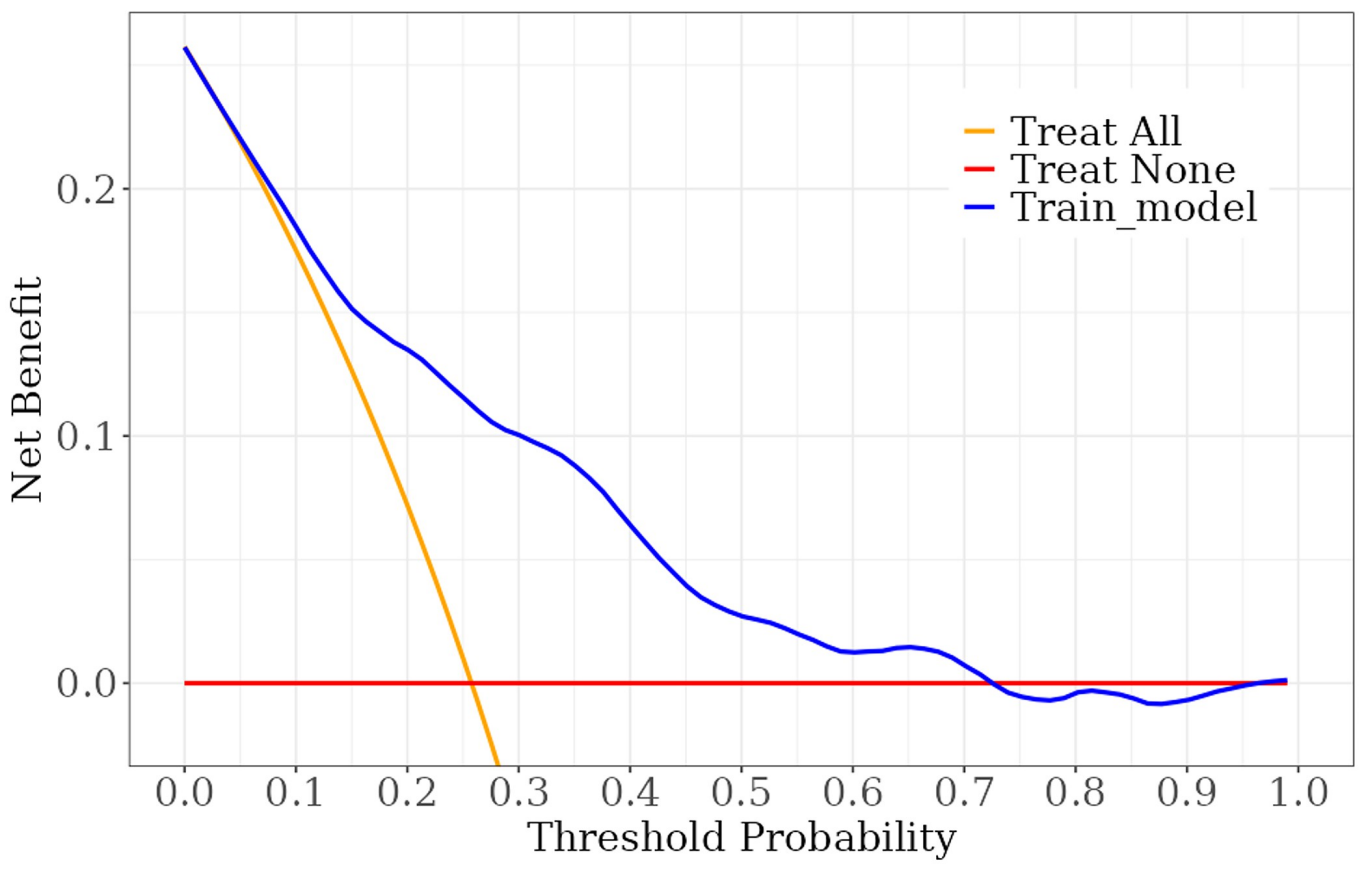


We constructed a nomogram model to predict recurrent vertebral fractures within 3 years after PVP based on the five risk factors identified by multivariate logistic regression analysis (Fig 2). By substituting these five risk factors into the nomogram model, surgeons can predict the probability of NVCF after PVP in patients with OVCF, and provide appropriate recommendations to different patients to prevent NVCF. The area under the curve (AUC) (0.781, 95%CI: 0.731–0.831) of the ROC curve suggested good discrimination of this model in training set (Fig 3) and in validation set (Fig 4), respectively. The calibration curve suggested good prediction accuracy between the actual and predicted probabilities in training set (Fig 5) and in validation set (Fig 6), respectively. The decision curve analysis (DCA) results showed that when the probability threshold was between 0.0452 and 0.8394 in training set (Fig 7) and between 0.0336 and 0.7262 in validation set (Fig 8), patients can benefit from using this model to predict NVCF within 3 years after PVP.

## Discussion

NVCF after PVP in patients with OVCF is common. It has been reported that 9.3% to 38.4% of patients develop NVCF after primary OVCF [14,15]. Therefore, it is necessary to identify the potential risk factors for NVCF in advance and take preventive measures to reduce this probability. Factors related to refractures at the primary fracture segments or new OVCF at other segments have been identified. A retrospective study of 147 patients by Rho et al. found that 27 patients (18.4%) had subsequent symptomatic NVCF, with a median time to new fracture of 70 days. Additionally, they found that lower BMD and discal cement leakage were risk factors for NVCF [17]. A retrospective study of 139 cases by Yang et al. found that a lower preoperative BMD, larger cement volume, balloon volume, recovery of vertebral height, and cement leakage were associated with an increased risk of compression fractures of the adjacent vertebral bodies after percutaneous kyphoplasty [18]. Recently, Zhang et al. found that older age, female sex, and smoking were risk factors for NVCF in patients with OVCF, whereas postoperative exercise and osteoporosis treatment were protective factors [19]. In addition, although not widely adopted, some studies have found a positive correlation between the HU values of CT and NVCF in patients with primary OVCF [14,20,21]. This study included 611 patients with OVCF. We found that mean age, smoking status, rate of Kümmell disease, postoperative minor trauma, multisegment fractures, and lack of anti-osteoporosis treatment were significantly higher in the NCVF than in the non-NVCF group within 3 years after PVP, and the BMD were significantly lower in the NCVF group. Multivariate logistic regression analysis suggested that older age, lower BMD, smoking, postoperative minor trauma, and lack of anti-osteoporosis therapy were risk factors for NVCF after PVP in patients with OVCF.

The association between lower BMD and NVCF is not difficult to explain as previous studies have reported similar results [15,17,18]. In addition, we found that the lack of anti-osteoporosis treatment was also a risk factor, as Zhang et al. found osteoporosis treatment to be a protective factor [19]. Therefore, standard anti-osteoporotic measures after PVP surgery should assume the same importance as the surgery itself, in order to reduce the probability of postoperative NCVF. Similar to Zhang et al. study, we found that older age was a risk factor for NCVF [19]. We found the probability of developing NVCF increases by approximately 8.4% for every 1 year of age increase in patients. In addition to possible poorer bone quality, older patients may have a higher incidence of accidental falls and poorer compliance with osteoporosis treatment. These factors may be associated with the development of new vertebral fractures. In addition, elderly patients with postoperative minor trauma, such as sudden weight bearing, are more likely to develop NVCF. However, vertebral fractures do not occur in normal adults with accidental falls, slipping, or sudden weight bearing. Accidental falls, slipping, and weight-bearing activities may be the absolute limit for patients with primary OVCF. Therefore, compared to younger OVCF patients, older OVCF patients should be informed and emphasized in more detail about their risk of developing NVCF to increase their emphasis on preventing NVCF. Given that minor postoperative trauma still increases the probability of NVCF, OVCF patients should not be careless after symptom improvement after PVP surgery, but should be extra careful to avoid postoperative trauma. In addition, we found that smoking was associated with new vertebral fractures. The probability of developing NVCF in patients who smoke after PVP surgery is approximately 1.761 times higher than in non-smoking patients. This is easy to explain, as many patients reported experiencing sudden back pain after a continuous cough while smoking and were subsequently diagnosed with new vertebral fractures. Therefore, smoking cessation is strongly recommended, as it may reduce the incidence of new vertebral fractures. Finally, we found the probability of NVCF increases by approximately 33.9% for every 1 unit decrease in BMD, and the probability of developing NVCF in patients without postoperative anti-osteoporosis treatment is approximately 2.214 times higher than that in patients with postoperative anti-osteoporosis treatment. Therefore, for patients after PVP, the standard procedure of anti-osteoporosis cannot be overemphasized, especially in clinical practice, we found that many patients no longer receive anti-osteoporosis treatment after pain relief after PVP.

Recently, researchers developed predictive models for NVCF after OVCF [25–28]. A recent retrospective study by Bian et al. found that 23.8% (69/292) of patients develop NVCF after PVP surgery, and they found four independent risk factors, including age, HU value, cement leakage, and thoracolumbar junction fracture, could predict NVCF [26]. Li et al. found that 15.1% (58/385) of OVCF patients develop NVCF after PVP surgery, and, they found that higher BMI, lower BMD, multisegment vertebral fractures, no previous anti-osteoporosis treatment, and steroid use were more likely to result in NVCF after PVP surgery [27]. A retrospective study by Gao et al. found that 37% (74/200) of OVCF patients developed NVCF after PVP surgery. Additionally, they found that > 7 days from injury to operation, high homocysteine levels, low osteocalcin levels, osteoporosis, lack of anti-osteoporosis treatment after surgery, operation method (PVP), and poor bone cement diffusion were independent risk factors for NVCF in middle-aged and elderly patients with OVCF after bone cement injection. They developed a predictive nomogram for NVCF based on these seven risk factors, which proved to have good predictive performance [28]. However, none of the three studies clearly indicated the follow-up time of the patients, and the number of cases in their study is limited, which may lead to less accurate conclusions or be unsuitable for specific clinical scenarios. In this study, we used five risk factors screened using a multivariate logistic regression model, to develop and validate a nomogram prediction model for predicting NVCF within 3 years after PVP for OVCF. The ROC and calibration curves in the training and validation sets suggested good prediction performance for this model. Additionally, the DCA results in the training and validation sets suggested that when the probability threshold was between 0.0452–0.8394 and 0.0336–0.7262, patients can benefit from using this model to predict NVCF within 3 years of PVP. By substituting these five risk factors into the nomogram model, surgeons can predict the probability of NVCF after PVP in patients with OVCF, and provide appropriate recommendations to different patients to prevent NVCF.

### Limitations

OVCF is influenced by many factors, and we could not consider and include all potential risk factors reported in previous studies. In addition, the number of patients included in this study was limited. Furthermore, the retrospective nature of this study may lead to potential biases in patient selection and data collection. In future, prospective studies with larger sample sizes are needed to develop predictive models. Furthermore, we only included patients who were followed up for 3 years. Longer follow-up is necessary to assess the longer-term risk of NVCF. Finally, we may not have detected 100% of patients with NVCF, as some patients who develop NVCF after PVP surgery may not have obvious pain and therefore did not undergo MRI or other examinations to confirm NVCF, or some patients may experience severe back pain again without relevant follow-up examinations. These patients may be unintentionally excluded from NVCF. In addition, the case population in this study was from a single center, which may have caused unavoidable bias. Finally, this prediction model lacks validation from external data such as OVCF patient data from other hospitals. Whether this prediction model is effective for more diverse population remains to be further explored.

## Conclusion

Older age, lower BMD, smoking, lack of anti-osteoporosis therapy, and postoperative minor trauma are risk factors for NVCF. We developed a nomogram prediction model based on five risk factors that can effectively predict NVCF within 3 years after PVP surgery. Postoperative smoking cessation, standard anti-osteoporosis treatment, and reduction in incidental minor trauma are necessary and effective means of reducing the incidence of NVCF.

## Supporting information

S1 Checklist Human participants research checklist. (DOCX)

S1 Raw data (XLSX)
